# The Relationship of Precursor Cluster Concentration in a Saturated Crystallization Solution to Long-Range Order During the Transition to the Solid Phase

**DOI:** 10.32607/actanaturae.11815

**Published:** 2023

**Authors:** M. A. Marchenkova, A. S. Boikova, K. B. Ilina, P. V. Konarev, Yu. V. Pisarevsky, Yu. A. Dyakova, M. V. Kovalchuk

**Affiliations:** Federal Scientific Research Centre “Crystallography and Photonics”, Russian Academy of Sciences, Moscow, 119333 Russian Federation; National Research Centre “Kurchatov Institute”, Moscow, 123182 Russian Federation

**Keywords:** oligomers, precursor clusters, crystallization, supersaturation, crystal growth

## Abstract

A model for the transition from disordered liquid state to the solid phase has been proposed based on establishing a correlation between the concentration of precursor clusters in a saturated solution and the features of solid phase formation. The validity of the model has been verified experimentally by simultaneously studying the oligomeric structure of lysozyme protein solutions and the peculiarities of solid phase formation from these solutions. It was shown that no solid phase is formed in the absence of precursor clusters (octamers) in solution; perfect monocrystals are formed at a small concentration of octamers; mass crystallization is observed with an increasing degree of supersaturation (and concentration of octamers); further increase in octamer concentration leads to the formation of an amorphous phase.

## INTRODUCTION

Transition from the liquid disordered state to the solid state is an important area of condensed state physics; it has been researched for many decades [1, 2]. A large number of experimental studies on transition to the solid phase have been conducted for substances such as synthetic, metallic, dielectric, semiconductor, organic, macromolecular (including proteins), etc. Solutions of known compounds morph into a solid phase upon reaching their supersaturated state. It has been established also that the supersaturation degree determines the structure of the resulting solid phase. Monocrystals are formed at low supersaturation degrees. A further increase in the supersaturation degree results in mass crystallization and transition to the amorphous phase. However, there is no generally accepted model for this transition.

The conventional crystallization mechanism, which was once considered valid for all systems, is gradually being replaced by the non-conventional mechanism, which is now believed to be the prevailing mechanism of crystallization from solution and other systems [2, 3, 4, 5, 6, 7, 8, 9]. According to the conventional crystal growth theory, any crystal grows through the attachment of new building units (atoms, ions, molecules, and their complexes) to its surface from the environment (solution, melt, vapor, and solid). Non-conventional crystallization models postulate that crystals can grow via the addition of not only single atoms, ions, and molecules to their surface, like in the conventional theory, but also by addition of solid phase blocks. It should be noted that these studies describe the resulting precursors, particles, dense liquid drops with an amorphous structure, as well as the essential features of a series of liquid-to-solid phase transition cases for the processes occurring at the scale of tens of nm and above. These works denote the need to study the processes that take place in saturated (crystallization) solutions at the nm and tens of nm scale.

For the past decades, crystallization solution structures at a scale of units and tens of nanometers have been studied using the methods of small-angle X-ray scattering (SAXS) and small-angle neutron scattering (SANS), as well as the method of molecular dynamics. Such studies were conducted on the saturated crystallization solutions of a series of proteins [10, 11, 12, 13, 14, 15] and potassium dihydrogen phosphate [9]. In these works, three-dimensional (3D) fragments were isolated from the crystallization structure of the compounds under study. The fragments could then be used for monocrystal formation. These ordered formations are found in saturated solutions of lysozyme (octamers [10, 11, 12, 13, 14, 15]), thermolysin (hexamers [16]), proteinase (dimers [17]), aminotransferase (dodecamers [18]), and potassium dihydrophosphate (octamers [19]), which was experimental confirmation of the hypothesis on the existence and structure of a precursor cluster in a crystallization solution. The use of molecular dynamics showed that this cluster is stable in a crystallization solution [20]. In particular, it has been shown for lysozyme that only protein dimers and octamers are present in a crystallization solution, while other oligomers (tetra-, hexa-, decamers, etc.) are unstable [21].

The dependence of the concentration of lysozyme dimers and octamers on the temperature and concentration of the solvent and precipitant has been studied in detail by SAXS and SANS. It has been confirmed for a wide range of crystallization solution parameters that a lysozyme solution contains protein monomers, dimers, and octamers, and that their ratio depends on the degree of the solution’s saturation [10, 11, 12, 13, 14, 15].

The current work represents a study of the relation between the precursor cluster concentration and the features of a solid phase formation exemplified by lysozyme. For this, we performed two series of experiments. We analyzed the lysozyme oligomeric structure (the ratio of monomer, dimer, and octamer concentrations) in ~60 lysozyme solutions with different precipitates by SAXS. The transition of the solutions to the solid phase was also studied (crystals were grown using the method of hanging drop vapor diffusion).

## EXPERIMENTAL

**Preparation of crystallization solutions of the lysozyme protein**

To prepare the solutions, Sigma-Aldrich chicken egg lysozyme was used (CAS# 12650-88-3, USA). Solutions from the crystallization kits NeXtal-Tubes- Classics-Suite 1 and NeXtal-Tubes-Classics-Suite 2 (QIAGEN®) and sodium chloride (CAS 7647-14-5, Helicon, Russia) were used as precipitants. Sodium acetate (CAS 6131-90-4, Sigma-Aldrich) and acetic acid (CAS 64-19-7, PanReac AppliChem) were used to prepare sodium acetate buffer. Hereinafter, the precipitant solutions from the crystallization kits will be referred to as CS1 No. and CS2 No., respectively, where No. stands for the solution number in the kit. Lysozyme and NaCl were dissolved in 0.2 M sodium acetate buffer (pH 4.5) prepared using Millipore ultrapure water (water resistance 18 MOhm × cm). Before mixing with the precipitant, the protein solution was centrifuged at 10,000 rpm for 10 min. The initial concentration of the protein stock solution was 80 mg/ml; the solution was then diluted with a buffer to the required concentration.

Prior to SAXS, the lysozyme and stock solutions of the precipitant were mixed in equal volumes.

**SAXS measurements**

SAXS measurements of lysozyme solutions on a P12 EMBL BioSAXS beamline with a PETRA III synchrotron radiation source (DESY, Hamburg, Germany). Samples with different compositions of precipitants from the CS1 and CS2 kits were measured on a P12 EMBL BioSAXS beamline with a PETRA III synchrotron radiation source (DESY, Hamburg, Germany) [22]. The X-ray energy was 10 keV (λ = 0.124 nm). Data were collected using a PILATUS 6M 2D pixel detector (Dectris, Switzerland) at a sample–detector distance of 3.0 m covering a scattering vector range of 0.02 < s < 7.0 nm^−^1 (s = 4πsin*θ*/*λ*, where 2*θ *is the scattering angle), which corresponds to a resolution of 300–0.9 nm in real space. Measurements were carried out using a special cell for SAXS samples, consisting of a horizontal temperature-controlled (temperature range of 278–323 K) quartz capillary with a wall thickness of 50 μm and a diameter of 1.7 mm, placed in a specialized stainless steel pod for measurements in vacuum. The test solution moved uniformly along the capillary; each time, the beam reached the same site at the capillary but a different part of the sample. A total of 20 measurements were made for each sample. The exposure time was 50 ms. The sample volume for each measurement was 40 μl. All measurements were carried out at a temperature of 20°C and lysozyme concentration of 20 mg/ml.

SAXS measurements of lysozyme solutions on a BM29 BioSAXS beamline with a ESRF synchrotron radiation source (Grenoble, France). Samples with different concentrations of the NaCl precipitant (range, 5–30 mg/ml) were measured on a BM29 BioSAXS beamline with an ESRF synchrotron radiation source (Grenoble, France). The X-ray energy was 12.4 keV. Data were collected using a Pilatus 1M 2D pixel detector (Dectris). The sample–detector distance was 2.8 m. The studied samples were placed in a special temperature-controlled robotic system [23] in 200-μl polystyrol cells, which were simultaneously heated. The samples were heated to 20°C and then maintained at that temperature. The solution from the cell automatically entered a quartz capillary with a diameter of 1.8 mm, which was used for measurements. The test solution moved uniformly along the capillary; each time, the beam reached the same site at the capillary but a different part of the sample. A total of 10 measurements were made for each sample. The exposure time was 1 s; beam cross-section at the sample was 400 μm^2^.

**SAXS data processing**

The signal from the buffer solution was averaged, subtracted from the data of protein solution scattering, and normalized to the protein concentration using the PRIMUS program of the ATSAS software package [24, 25]. Experimental curves of scattering intensity I(s) were obtained for the protein solutions in different conditions. The addition of precipitants to the lysozyme solution in some specific conditions changes the oligomeric composition of the solution in a way that, in addition to monomeric particles, multimers (oligomers of a higher order: dimers, tetramers, hexamers, and octamers) are formed. For this reason, data were analyzed with account of the presence of several components in the system. After primary data processing, experimental SAXS curves were processed using the OLIGOMER software [25] to determine the volume fractions of monomers and oligomers of various orders. Theoretical curves of oligomeric components were calculated using the CRYSOL software [26]. The crystallographic structure of lysozyme (PDB ID: 4WLD) was used as a monomeric component, while dimer, tetramer, hexamer, and octamer models were obtained using the technique described in [10]. The fit quality χ^2^ was evaluated by minimizing the discrepancy between the experimental data and theoretical model approximations using the formula from [14].

**Lysozyme crystallization**

The stock solutions prepared for measurements by SAXS on a P12 EMBL BioSAXS beamline (DESY, Hamburg, Germany) were also used for lysozyme crystallization. The method of hanging drop vapor diffusion and a Mosquito-LCP crystallization robot (EMBL, Hamburg, Germany) were used for crystallization. The volume of each drop was 200 nl (100 nl of the protein stock solution + 100 nl of the stock precipitant solution). Crystals were grown in the automated imaging system ROCK IMAGER at 19°C. The system makes it possible to observe the growth of protein crystals and take photographs of drops during a long period of time (at day 0 (immediately after loading the crystallization plate) and then on days 1, 3, 7, 14, 28, 54, and 84). The same solutions from the kits NeXtal-Tubes-Classics-Suite 1 and NeXtal-Tubes- Classics-Suite 2 were used as precipitants similar to the SAXS analysis of the solutions. Lysozyme was crystallized at two concentrations: 20 and 40 mg/ml. We would like to note that the solutions were analyzed by SAXS at the same concentration (20 mg/ml).

## RESULTS AND DISCUSSION

As mentioned in the Introduction, crystallization can be described as a phase process of liquid-to-solid phase transition. Therefore, crystallization is a three-stage process (two-step crystallization): the solution is initially in liquid phase, then the intermediate phase takes shape, followed by the formation of the solid phase at the final stage.

**The effect of the precipitant concentration on the supersaturation degree and its dependence on octamer concentration**

A number of factors determine the supersaturation degree, which include both physical (e.g. temperature) and chemical parameters (concentrations of the precipitant and protein in the solution, the chemical composition of the solution, and the precipitant type). In this work, we studied the effect of the precipitant type and concentration on the composition of the intermediate phase (the content of monomers, dimers, and octamers in it). The results are presented in Table 1.

**Table 1:** Oligomer composition of the lysozyme crystallization solution (volume fractions of monomers, dimers, and octamers) using NaCl as the precipitant, obtained by SAXS

NaCl concentration, mg/ml	R_g_, A	Monomer, %	Dimer, %	Octamer, %	χ^2^
30	21.0	85.6	10.3	4.1	1.46
25	20.7	87.3	8.9	3.8	1.41
20	20.3	88.8	7.7	3.5	1.30
15	19.5	92.0	5.1	2.9	1.43
5	15.5	95.2	4.5	0.3	1.78

Note. Measurements were carried out at 20°C. Rg – radius of gyration. χ2 – fit quality.

According to the equation (2) from [27], lysozyme solubility (Cs) and NaCl concentration (CNaCl) in sodium acetate buffer (pH 4.6) at 293 K are related through the following relationship:

C_s_ = –0.0016C^3^_NaCl_ + 0.2146C^2^_NaCl_ – 9.6437C_NaCl_ + 148.06

The relation between supersaturation (σ), lysozyme solubility (C_s_), and concentration (C) in the solution is described by the following equation: σ = C / C_s_ [24].

**Fig. 1:**
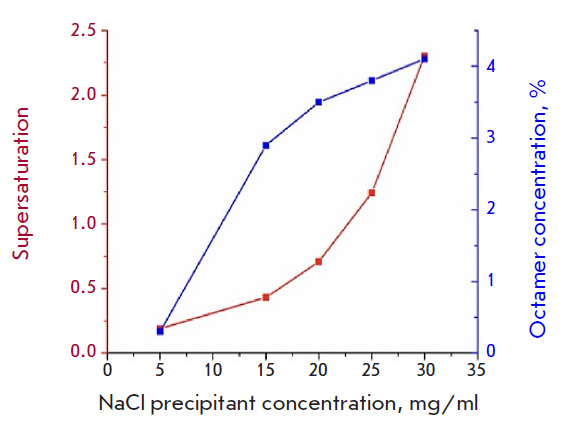
Lysozyme supersaturation (red curve) and octamer volume fraction (blue curve) in a lysozyme solution at different NaCl concentrations

The resulting curve of lysozyme solution supersaturation and octamer concentration versus NaCl concentration in the solution is presented in Fig. 1.

Both supersaturation and the volume fraction of octamers grow with an increase in the NaCl concentration, with supersaturation increasing not gradually but almost exponentially, starting at a given time point. The point of the lowest supersaturation and highest solubility at an NaCl concentration of 5 mg/ml corresponds to the lowest volume fraction of octamers (0.3%), which makes crystallization unlikely.

**Table 2:** Oligomer composition of lysozyme crystallization solutions with precipitators from the CS1 and CS2 crystallization kits by SAXS

№	Precipitant	Rg, Å	Dimer, %	Octamer, %	χ^2^	Crystallization result	Precipitant solution composition
1	CS1 1	14.3	0	0	3.26	–	0.01 M cobalt chloride 0.1 M sodium acetate pH 4.6 1.0 M 1,6-hexanediol
2	CS1 10	15.5	9.3	0	1.37	–	0.2 M magnesium chloride 0.1 M HEPES HEPES sodium salt pH 7.5 30% (v/v) isopropanol
3	CS1 11	15.0	5.4	0	1.25	–	0.2 M ammonium acetate 0.1 M Tris-HCl pH 8.5 30% (v/v) isopropanol
4	CS1 14	14.3	0	0	6.97	–	25% (v/v) ethylene glycol
5	CS1 15	14.3	0	0	2.24	–	0.02 M calcium chloride 0.1 M sodium acetate pH 4.6 30% (v/v) MPD (2-methyl-2,4-pentanediol)
6	CS1 16	14.5	1.7	0	1.18	–	0.2 M sodium chloride 0.1 M sodium acetate pH 4.6 30% (v/v) MPD
7	CS1 17	16.3	18.4	0	1.2	–	0.2 M ammonium acetate 0.1 M trisodium citrate pH 5.6 30% (v/v) MPD
8	CS1 2	16.3	18.4	0	1.69	–	0.1 M trisodium citrate pH 5.6 2.5 M 1,6-hexanediol
9	CS1 21	15.6	10.9	0	1.43	–	0.2 M ammonium phosphate 0.1 M Tris pH 8.5 50 %(v/v) MPD
10	CS1 23	14.3	0	0	2.17	–	0.1 M Tris pH 8.5 25% (v/v) tert-butanol
11	CS1 25	16	14.9	0	1.71	–	0.4 M ammonium phosphate
12	CS1 3	14.3	0	0	1.08	–	0.2 M magnesium chloride 0.1 M Tris pH 8.5 3.4 M 1,6-hexanediol
13	CS1 51	14.3	0	0	7.41	–	35% (v/v) dioxane
14	CS1 61	14.5	1.2	0	1.15	–	0.2 M magnesium formate
15	CS1 18	14.3	0	0	1.17	–	0.2 M magnesium acetate 0.1 M sodium cacodylate pH 6.5 30% (v/v) MPD
16	CS1 20	17.1	25.9	0.1	1.73	–	0.5 M ammonium sulfate 0.1 M HEPES pH 7.5 30% (v/v) MPD
17	CS1 24	17.6	33.5	0.1	3.06	–	0.1 M trisodium citrate pH 5.6 35% (v/v) tert-butanol
18	CS1 9	18	39.3	0.1	3.76	–	0.2 M trisodium citrate 0.1 M sodium cacodylate pH 6.5 30% (v/v) isopropanol
19	CS2 13	14.3	0	0.1	1.35	–	0.3 M magnesium formate 0.1 M Bis-Tris pH 5.5
20	CS1 56	17.5	20.8	0.6	1.14	–	0.1 M HEPES pH 7.5 20% (v/v) Jeffamine M-600
21	CS1 35	17.1	14.2	0.6	1.18	–	1.0 M imidazole pH 7
22	CS1 6	16	3.9	0.6	1.08	–	0.2 M calcium chloride 0.1 M sodium acetate pH 4.6 20% (v/v) isopropanol
23	CS1 8	18.1	29.6	0.7	1.76	–	0.2 M trisodium citrate 0.1 M HEPES sodium salt pH 7.5 20% (v/v) isopropanol

Note. Samples are arranged in increasing order of octamer volume fraction (0–0.7%).

**Table 3:** Oligomer composition of lysozyme crystallization solutions with precipitators from the CS1 and CS2 crystallization kits by SAXS

№	Precipitant	Rg, Å	Dimer, %	Octamer, %	χ^2^	Crystallization result	Precipitant solution composition
24	CS1 39	17.4	14.8	0.9	1.32	Crystal	0.05 M cadmium sulfate 0.1 M HEPES pH 7.5 1.0 M sodium acetate
25	CS2 17	18.5	28.9	1	1.08	Aggregation	1.26 M sodium phosphate 0.14 M potassium phosphate
26	CS1 62	18.3	25.1	1	1.25	Crystal	0.1 M MES pH 6.5 1.6 M magnesium sulfate
27	CS1 46	18.3	24.5	1.1	1.17	Crystal	0.1 M HEPES sodium salt pH 7.5 0.8 M sodium phosphate 0.8 M potassium phosphate
28	CS1 50	18.3	20.5	1.2	1.28	Aggregation	1.6 M ammonium sulfate 0.1 M MES pH 6.5 10% (v/v) dioxane
29	CS1 27	19.2	29.1	1.6	1.26	Crystal	0.1 M Tris-HCl pH 8.5 2.0 M ammonium phosphate
30	CS1 37	19.1	24.5	1.7	1.28	Crystal	0.1 M HEPES sodium salt pH 7.5 0.8 M K/Na tartrate
31	CS1 58	19.5	28	1.9	1.28	–	0.01 M nickel chloride 0.1 M Tris pH 8.5 1.0 M lithium sulfate
32	CS1 28	19.2	19.2	2	1.2	Crystal	0.1 M HEPES pH 7.5 2.0 M ammonium formate
33	CS1 59	19.6	27.7	2.1	1.34	Aggregation	0.1 M HEPES sodium salt pH 7.5 1.5 M lithium sulfate
34	CS1 32	19.7	23.6	2.3	1.25	Aggregation	0.1 M sodium chloride 0.1 M HEPES pH 7.5 1.6 M ammonium sulfate
35	CS1 22	21.1	66.8	2.5	12.72	Crystal	0.1 M HEPES pH 7.5 70 % (v/v) MPD
36	CS2 22	19.9	22.8	2.5	1.18	Crystal	0.8 M succinic acid pH 7.0
37	CS1 33	20.1	23.9	2.7	1.26	Aggregation	0.01 M cobalt chloride 0.1 M MES pH 6.5 1.8 M ammonium sulfate
38	CS2 18	20.5	32.6	2.9	1.14	Aggregation	0.49 M sodium phosphate 0.91 M potassium phosphate
39	CS1 4	20.7	25.1	3.3	1.22	Aggregation	2.0 M ammonium sulfate 5% (v/v) isopropanol
40	CS1 57	20.8	27.6	3.4	1.27	Aggregation	0.5 M ammonium sulfate 0.1 M sodium citrate pH 5.6 1.0 M lithium sulfate
41	CS1 30	21.1	24.6	3.8	1.23	Aggregation	0.1 M Tris-HCl pH 8.5 2.0 M ammonium sulfate
42	CS2 19	21.4	29.8	4	1.06	Aggregation	0.056 M sodium phosphate 0.91 M potassium phosphate
43	CS1 29	21.3	23.7	4	1.09	Aggregation	0.1 M ammonium acetate pH 4.6 2.0 M ammonium sulfate
44	CS1 60	21.4	23	4.2	1.18	–	0.1 M BICINE pH 9.0 2.0 M magnesium chloride
45	CS1 31	21.5	24.6	4.3	1.2	Aggregation	2.0 M ammonium sulfate
46	CS1 47	21.6	22.4	4.4	1.13	Crystal	0.1 M sodium acetate pH 4.6 2.0 M sodium formate

Note. Samples are arranged in increasing order of octamer volume fraction (0.9–4.4%).

**Table 4:** Oligomer composition of lysozyme crystallization solutions with precipitators from the CS1 and CS2 crystallization kits by SAXS

№	Precipitant	Rg, Å	Dimer, %	Octamer, %	χ^2^	Crystallization result	Precipitant solution composition
47	CS1 43	18	0	4.9	23.69	Denaturation	0.1 M HEPES pH 7.5 4.3 M sodium chloride
48	CS2 21	22.3	35.9	5.1	1.27	Denaturation	1.8 M ammonium citrate pH 7.0
49	CS1 42	22.2	24.4	5.2	1.15	Aggregation	0.1 M sodium phosphate 0.1 M potassium phosphate 0.1 M MES pH 6.5 2.0 M sodium chloride
50	CS1 41	22.8	21.3	6.2	1.12	Aggregation	0.1 M sodium acetate pH 4.62 M sodium chloride
51	CS2 24	23.4	23.9	7.1	1.13	Denaturation	2.8 M sodium acetate pH 7.0
52	CS1 34	23.5	25.1	7.4	1.17	Denaturation	0.2 M K/Na tartrate 0.1 M trisodium citrate pH 5.6
53	CS1 44	26.2	15.1	13.5	1.17	Denaturation	0.1 M HEPES sodium salt pH 7.5 1.4 M trisodium citrate
54	CS1 45	26.8	20.5	16	1.42	Denaturation	1.6 M trisodium citrate pH 6.5
55	CS1 48	28.2	0	21.1	18.85	Denaturation	4.0 M sodium formate

Note. Samples are arranged in increasing order of octamer volume fraction (4.9–21.1%).

The volume fraction of octamers in the solution increases from 2.9 to 4.1% with an increase in the NaCl concentration (from 15 to 30 mg/ml). At the same time, an increase in the precipitant concentration leads to a rapid rise in the supersaturation degree, which indicates the instability of this state, when even a slight change in the environment can significantly affect the solution supersaturation degree. In other words, in the region where the volume fraction of octamers exceeds 4%, the probability of aggregation and occurrence of crystals would decrease, while the probability of aggregation and formation of an amorphous precipitate, on the contrary, should increase.

**Comparison of results of the transition to a solid phase with octamer concentrations**

An analysis of the data obtained by SAXS (oligomeric composition of solutions, radius of gyration (R_g_), and fit quality χ^2^) of the crystallization solutions with the CS1 and CS2 precipitant kits is presented in Table 2, Table 3, Table 4. For each case, the Tables provide the chemical compositions of the precipitants and crystallization results, which, in our case, could be one of the four variants below:

• clear drops (no crystal, result "–");

• the crystal grew at the study concentration of 20 mg/ml in at least one drop (result "crystal");

• aggregation (mass crystallization, result "aggregation"); and

• denaturation (amorphous precipitant, result "denaturation").

There were 55 such cases out of 67 studied solutions.

In the presence of the remaining 12 precipitants (CS1 13, CS1 55, CS2 16, CS2 14, CS2 15, CS1 36, CS1 26, CS1 54, CS1 38, CS1 40, CS1 12, and CS1 52), no crystal growth was noted in the solution with a protein concentration of 20 mg/ml. However, a crystal was observed at a concentration of 40 mg/ml in the solution with the same precipitant. In the vast majority of cases, a crystal was observed either after a very long period of time (28–56 days) or in one out of three drops.

It is extremely important to note that, in all the cases when no octamers were present in the solution (only monomers and dimers), no crystals were found. In addition, neither aggregation nor denaturation was observed (all three drops remained visually clear during the whole period of exposure). Taking into account the previous studies by SANS [13] and the discovered growth step height (110) for tetragonal crystals (6 nm) [28, 29], we can state that the **formation of lysozyme octamers is the key stage of lysozyme crystallization **[10], while dimers cannot be the key element of the protein crystal.

We can distinguish three general cases describing crystallization solutions with different compositions and corresponding to different regions in the phase diagram:

1) only monomers are present in the solution (the lowest point of the unsaturated region);

2) only monomers and dimers are found in the solution (unsaturated region/approaching saturation); and

3) monomers, dimers, and octamers are observed in the solution, with the octamer concentration changing depending on the supersaturation degree as follows (four intervals in total):

1. 0–1%: crystals or other solid formations are absent in the solution;

2. 1–5%: monocrystal growth and aggregation are observed;

3. 5–7%: either aggregation or denaturation occurs (amorphous formation); and

4. > 7%: only denaturation is noted.

**Fig. 2:**
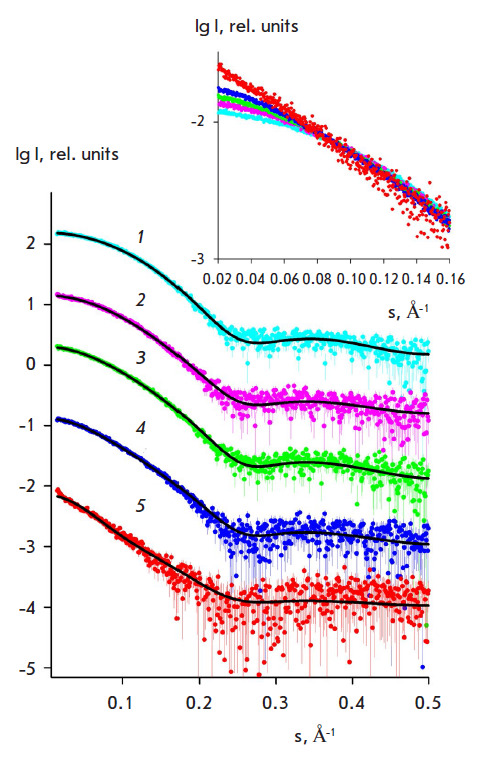
Experimental SAXS curves (colored lines) for lysozyme crystallization solutions (black lines) and theoretical approximations using a mixture of oligomers calculated using the OLIGOMER program for the following solutions: *1 *– lysozyme with CS1 18 precipitant (monomers only, no crystal), *2 *– lysozyme with CS1 17 precipitant (dimers and monomers, no crystal), *3 *– lysozyme with CS1 31 precipitant (monomers, dimers, octamers, and crystal), *4 *– lysozyme with CS1 31 precipitant (monomers, dimers, octamers, and aggregation), *5 *– lysozyme with CS1 45 precipitant (monomers, dimers octamers, and denaturation). The curves are shifted along the vertical axis for better visualization

Figure 2 shows five experimental SAXS curves for a lysozyme solution with the precipitants CS1 18 (No. 15 in Table 2), CS1 17 (No. 7 in Table 2), CS1 28 (No. 32 in Table 3), CS1 31 (No. 45 in Table 3), and CS1 45 (No. 54 in Table 4). These curves present different cases of oligomeric composition of the lysozyme solution and crystallization results. In the case where CS1 18 was used, when only monomers were observed in the solution, clear drops were noted (neither crystals nor other solid phases were formed). For CS1 17 (monomers and dimers were observed, while octamers were not found in the solution), a clear drop was also noted (result "–"). The use of CS1 28 (monomers, dimers, and octamers were observed in the solution) resulted in crystal growth (result "crystal"). The precipitants CS1 31 and CS1 45 led to the formation of monomers, dimers, and octamers in the crystallization solutions, as well as aggregation and denaturation (CS1 31 and CS1 45, respectively).

**Dependence of the probability of crystal growth on the precursor cluster concentration in the crystallization solution**

Preparation of the crystallization solution of the protein can result in several evolutionary pathways depending on the supersaturation degree:

1) no precursor clusters form in the protein solution after addition of the precipitant: i.e., there is no intermediate and, therefore, solid phase, including monocrystals (in addition to monomers, dimers may be also present in the solution, (unsaturated region, approaching saturation));

2) precursor clusters form in the solution, the intermediate phase forms, with further transition to crystal growth;

3) the degree of solution supersaturation becomes so high that the precursor cluster concentration increases to the point where aggregation takes place (the state that can further evolve into a crystal); and

4) the supersaturation degree exceeds its limit, and the protein in the solution transforms into an amorphous state, when partial denaturation can occur.

**Table 5:** Probabilities calculated for the three cases: crystal growth at 20 mg/ml, occurrence of visible aggregation (mass crystallization), and occurrence of amorphous precipitation/denaturation

Octamer fraction (rounded to the nearest whole number), %	Total No. of cases	Crystal		Aggregation		Denaturation	
		Success	Probability	Success	Probability	Success	Probability
0	24	0	0	0	0	0	0
1	12	3	0.25	2	0.16667	0	0
2	7	3	0.42857	2	0.28571	0	0
3	8	2	0.25	4	0.5	0	0
4	6	1	0.16667	4	0.66667	0	0
5	3	0	0	1	0.33333	2	0.66667
6	2	0	0	1	0.5	0	0
7	2	0	0	0	0	2	1
14	1	0	0	0	0	1	1
16	1	0	0	0	0	1	1
21	1	0	0	0	0	1	1

Comparison of the results of a measuring of octamer concentrations and the peculiarities of lysozyme transition from solution to the solid phase denotes a relationship between them. In the absence of octamers in the solution (only monomers and dimers) at an octamer concentration < 1%, neither lysozyme monocrystals nor any other solid and amorphous forms were observed. Crystallization and aggregation are noted in a octamer concentration interval of 1–5%, with monocrystals forming mainly in the range of an octamer concentration of 2–3%. An increase in the octamer concentration to 5–7% leads to aggregation and denaturation (amorphous formation), while a further increase above 7% results in protein denaturation only (formation of amorphous precipitates).

In this regard, the data were converted into a different format. We considered the probability of successful crystal growth at different octamer fractions in the solution and rounded the octamer fractions to the nearest whole number (using the standard built-in Excel function). We considered the following cases and calculated their probability: 1 – crystal growth at 20 mg/ml; 2 – occurrence of visible aggregation (mass crystallization); and 3 – formation of an amorphous precipitate (denaturation). Therefore, the probability was calculated as the ratio of successful cases (when one of the abovementioned variants is observed in the drop) to the number of cases with the same octamer fraction (Table 5).

**Fig. 3:**
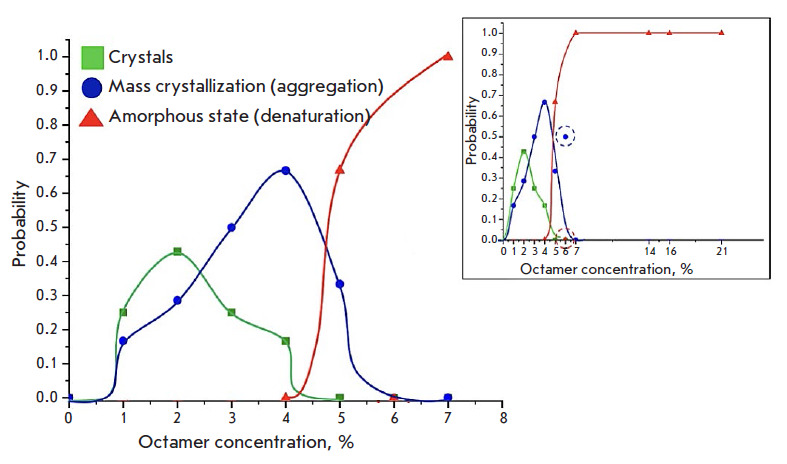
General patterns of the changes in the probability of lysozyme crystal growth, aggregation, and denaturation as a function of the integer fraction of octamers (21% magnification of the interval of the volume fraction of octamers in the inset), for different cases of condensed phase formation: crystals (green line), mass crystallization (blue line), and amorphous precipitate/denaturation (red line)

The highest probability of lysozyme crystal formation coincides with an octamer concentration range of 2–3% (Fig. 3). Neither crystal growth nor aggregation is observed for an octamer concentration of > 7%; the probability of denaturation in this case is 1. The general correlation between the formation of a given condensed phase as a result of crystallization and octamer concentration in the solution is as follows. The probability of any solid phase formation is zero (0) for a volume fraction of octamers of 0–1%. A higher octamer volume fraction corresponds to a higher probability of crystal formation and aggregation. Furthermore, the highest probability of crystal formation falls on an octamer concentration of 2%, while the maximum probability of aggregation shifts to a higher octamer concentration and stands at 4%. The probability of amorphous phase formation reaches its maximum at an octamer concentration of > 7%.

We would like to note that two points corresponding to an octamer fraction rounded to 6% were excluded from the general probability curves for aggregation and denaturation in Fig. 3. The reason why these points did not fit into the curves may have to do with the small statistical sample (only two cases).

In the section "The effect of the precipitant concentration on the supersaturation degree and its dependence on the octamer concentration", where we compared octamer concentrations at different NaCl concentrations, the supersaturation degree, and solubility, we stated that an increase in the NaCl concentration (from 15 to 30 mg/ml) results in an increase in the proportion of octamer fractions in the solution ranging from 2.9 to 4.1%. Measurements of supersaturation at different NaCl concentrations demonstrated that, in the region of octamer concentration of > 4%, the probability of monocrystal formation should decrease, while the probability of aggregation and amorphous state formation should increase. This pattern is observed in Fig. 3: the probability of crystal growth decreased to 0, while the probability of aggregation and denaturation began to increase at an octamer concentration of > 4%.

Based on a comparison of the obtained graphs with regions in the phase diagram, we can state that an octamer concentration of 0% (either monomers only or both dimers and monomers are present in the solution) corresponds to the unsaturated region in the phase diagram. An increase in the octamer volume fraction results in an increase in the supersaturation degree, and an octamer concentration range of 1–5% corresponds to the region of nucleation. An octamer concentration of >5% corresponds to the region of precipitation.

## CONCLUSIONS

Simultaneous measurement of oligomer concentrations and solid phase formation in 67 lysozyme solutions led us to the following conclusions:

• no crystals form at low octamer concentrations ( < 0.8%);

• at an octamer concentration in the range of 0.7–4%, either individual crystals form or mass crystallization (aggregation) takes place, with the aggregation probability increasing with increasing precursor cluster concentration (solution supersaturation degree); and

• the probability of amorphous (denatured) phase formation shoots up, starting from an octamer concentration of 6%.

**Fig. 4:**
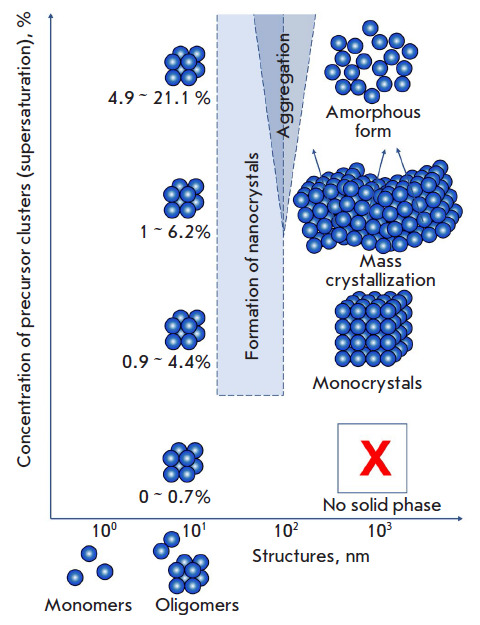
Polymorphism of the structures formed during crystallization depending on the concentration of precursor clusters as exemplified by the lysozyme protein under tetragonal syngony crystallization conditions

Based on our previous results on the link between the octamer concentration in a lysozyme solution at the initial crystallization stage and the crystallization results (formation of either monocrystals, aggregates, or an amorphous state), we can state that crystallization at the initial stage depends on the type of final solid phase the protein transforms into (Fig. 4). The obtained results make it possible to compare the processes under way in the region of 3–20 nm, which is the intermediate phase when precursor clusters form from 3D clusters, with the results of the transition to the solid phase (for sizes of 1–10 μm).

As noted above, the concentration of precursor clusters determines the solid phase type. Transition from clusters to crystals and an amorphous state was not studied in this work. This issue remains the least studied to date. It has been known since Faraday’s times that a supercooled liquid can exist for an indefinite period of time without transitioning to a solid state. This process is usually initiated by the temperature and concentration gradients, the addition of foreign objects that can act as seeding agents, etc. In this work, we performed crystallization at the concentration gradient, while the solid phase formed almost three months after the preparation of the solution.

Nevertheless, the result of this study makes it possible to predict the solid phase type at an early stage of solution (melt) preparation by measuring (or calculating) the concentration of the precursor clusters.

In addition, the nucleation process [30] remains unstudied. However, the obtained results can be useful in furthering our understanding of this process.

## Abbreviations

SAXS: small-angle X-ray scattering
SANS: small-angle neutron scattering
